# A Recent Global Selective Sweep on the *age-1* Phosphatidylinositol 3-OH Kinase Regulator of the Insulin-Like Signaling Pathway Within *Caenorhabditis remanei*

**DOI:** 10.1534/g3.114.010629

**Published:** 2014-04-11

**Authors:** Richard Jovelin, Jennifer S. Comstock, Asher D. Cutter, Patrick C. Phillips

**Affiliations:** * Institute of Ecology and Evolution, University of Oregon, Oregon 97403; † Department of Ecology and Evolutionary Biology, University of Toronto, Ontario M5S 3B2, Canada

**Keywords:** selective sweep, insulin pathway, *Caenorhabditis*, aging, molecular evolution

## Abstract

The discovery that genetic pathways can be manipulated to extend lifespan has revolutionized our understanding of aging, yet their function within natural populations remains poorly characterized. In particular, evolutionary theories of aging predict tradeoffs in resource investment toward somatic maintenance *vs.* reproductive output that should impose strong natural selection on genetic components that influence this balance. To explore such selective pressure at the molecular level, we examine population genetic variation in the insulin-like signaling pathway of the nematode *Caenorhabditis remanei*. We document a recent global selective sweep on the phosphoinositide-3-kinase pathway regulator, *age-1*, the first life-extension gene to have been identified. In particular, we find that *age-1* has 5−20 times less genetic variation than any other insulin-like signaling pathway components and that evolutionary signatures of selection center on the *age-1* locus within its genomic environment. These results demonstrate that critical components of aging-related pathways can be subject to shifting patterns of strong selection, as predicted by theory. This highly polymorphic outcrossing species offers high-resolution, population-level analyses of molecular variation as a complement to functional genetic studies within the self-reproducing *C. elegans* model system.

It is clearly advantageous for organisms to live and continue reproducing for as long as possible. The evolutionary explanation for why organisms instead tend to age and die derives from the fact that the high reproductive value of offspring produced early in life weakens the relative strength of selection against deleterious mutations acting later in life. This can result either in the accumulation of mutations with late-onset, age-specific effects (mutation accumulation; Medawar 1952) or the preferential fixation of alleles with favorable effects early in life, even if they have negative consequences later in life (antagonistic pleiotropy; Willams 1957). Under either of these scenarios, we might expect aging to result from the accumulation of genetic problems in a diverse set of biological systems. It was therefore somewhat surprising when *age-1*, the first mutation shown to extend life span (in this case in the nematode *Caenorhabditis elegans*), was described by Friedman and Johnson (1988). Even more surprising was the fact that *age-1* is part of the larger genetic pathway controlling insulin signaling (Figure 1) in which disruption of multiple components, most notably the *daf-2* insulin receptor (Kenyon *et al.* 1993), can also lead to life extension in nematodes and a wide variety of other animals (Garofalo 2002; Barbieri *et al.* 2003; Kenyon 2005; Broughton and Partridge 2009). The most likely explanation for the conserved effects of this pathway on longevity is that it mediates a physiological switch point that governs a trade-off between investment in reproduction and investment in the response to stress (*e.g.*, starvation) (Kirkwood 2002). Indeed, the insulin-signaling pathway satisfies the structural expectations of the antagonistic pleiotropy model of aging as longevity mutations in *age-1* and *daf-2* show a fitness cost under nutrient stress (Walker *et al.* 2000; Jenkins *et al.* 2004). As such, we would expect the pattern of selection on the regulation of the insulin-signaling pathway to vary over time with shifts in the environment and with changes in the demographic structure of populations. This expectation is further motivated by the pattern of selection for the longevity gene *methuselah* (*mth*) in *Drosophila*. Mutants for the G-protein coupled receptor *mth* have increased lifespan but also show a trade-off between longevity and reproduction under some circumstances (Mockett and Sohal 2006). Moreover, the gene *mth* is adaptively evolving (Schmidt *et al.* 2000), and allelic diversity in *mth* among populations coincides with clinal variation in longevity (Schmidt *et al.* 2000; Duvernell *et al.* 2003) and contributes to genetic differences in lifespan (Paaby and Schmidt 2008), further implicating natural selection acting on lifespan and on genetic variation at this locus. However, the correlation between variation in lifespan and allelic variation at *mth* could differ among populations and/or depend on specific environments (Sgro *et al.* 2013).

**Figure 1:**
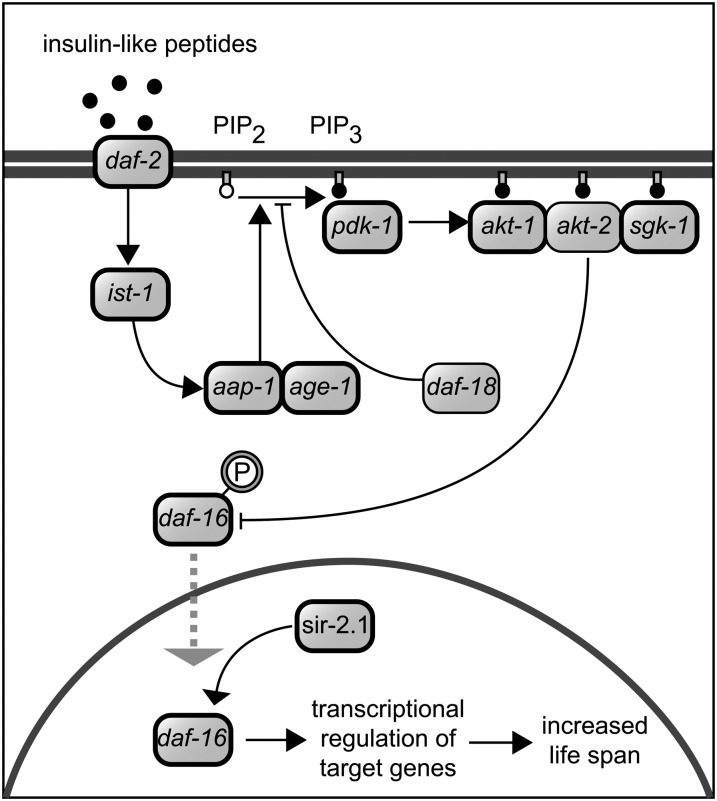
Core components of the insulin-like signaling pathway in *C. elegans*. Genes examined in this study are indicated by a bold outline (clear *C. remanei* homologs of *akt-2* and *daf-18* could not be identified).

Nevertheless, the number of studies investigating patterns of selection in genes involved in trade-offs between lifespan and reproduction is limited. A rational aim would be to look for evidence of this kind of selection within *C. elegans*, the species in which the majority of the aging-related mutations have been isolated. However, the natural ecology of *C. elegans* is not well defined, and its population genomic structure makes it difficult to use DNA sequence variation to make inferences about the evolutionary forces generating a phenotypic variation. In particular, linkage disequilibrium (LD) spans whole chromosomes (Cutter 2006; Rockman and Kruglyak 2009; Andersen *et al.* 2012), suggesting that both background selection and selective sweeps are likely to perturb genetic variation and nucleotide sites far away from the site under selection (Gaertner and Phillips 2010). For example, the vast majority of variation in gene transcript level within the species appears to be well described as a function of background selection operating in genomic regions of low recombination (Rockman *et al.* 2010). Moreover, the total amount of genetic variation within this species, which appears to be largely tied up within a few dozen haplotypes (Rockman and Kruglyak 2009; Andersen *et al.* 2012), is very low and does not reflect geographic structure, perhaps reflecting fairly recent dispersal of *C. elegans* around the world (Phillips 2006).

The pattern of nucleotide variation within *C. elegans* differs starkly with the gonochoristic or obligately outcrossing species of the genus. For example, *C. remanei* is a temperate species that lives in association with terrestrial isopods and displays ~20-fold more sequence polymorphism than *C. elegans* (reviewed in Cutter *et al.* 2013). LD also breaks down very rapidly within the species (on the order of a few hundred base pairs; Cutter *et al.* 2006; Dey *et al.* 2012), making it ideal for high-resolution mapping of recent evolutionary changes. The recent discovery of a near outgroup for *C. remanei*, *C*. sp. 23 (Dey *et al.* 2012) is particularly valuable in this regard because it is now possible to analyze patterns of genetic divergence more accurately, which has been heretofore problematic in *Caenorhabditis* because the large degree of divergence among currently sequenced species tends to lead to saturation of neutral sites in the genome. Here, we build on the functional knowledge generated within *C. elegans* and take advantage of the population genetic strengths of *C. remanei* to examine patterns of sequence variation across the entire insulin-like signaling (IS) pathway. We find a clear genomic footprint of a recent selective sweep on one pathway component (*age-1*), suggesting that the shifting pattern of natural selection on genes influencing the balance between investment in early and late life function predicted by theory can be observed within this species.

## Material and Methods

### Identification of orthologs

**Figure 2:**
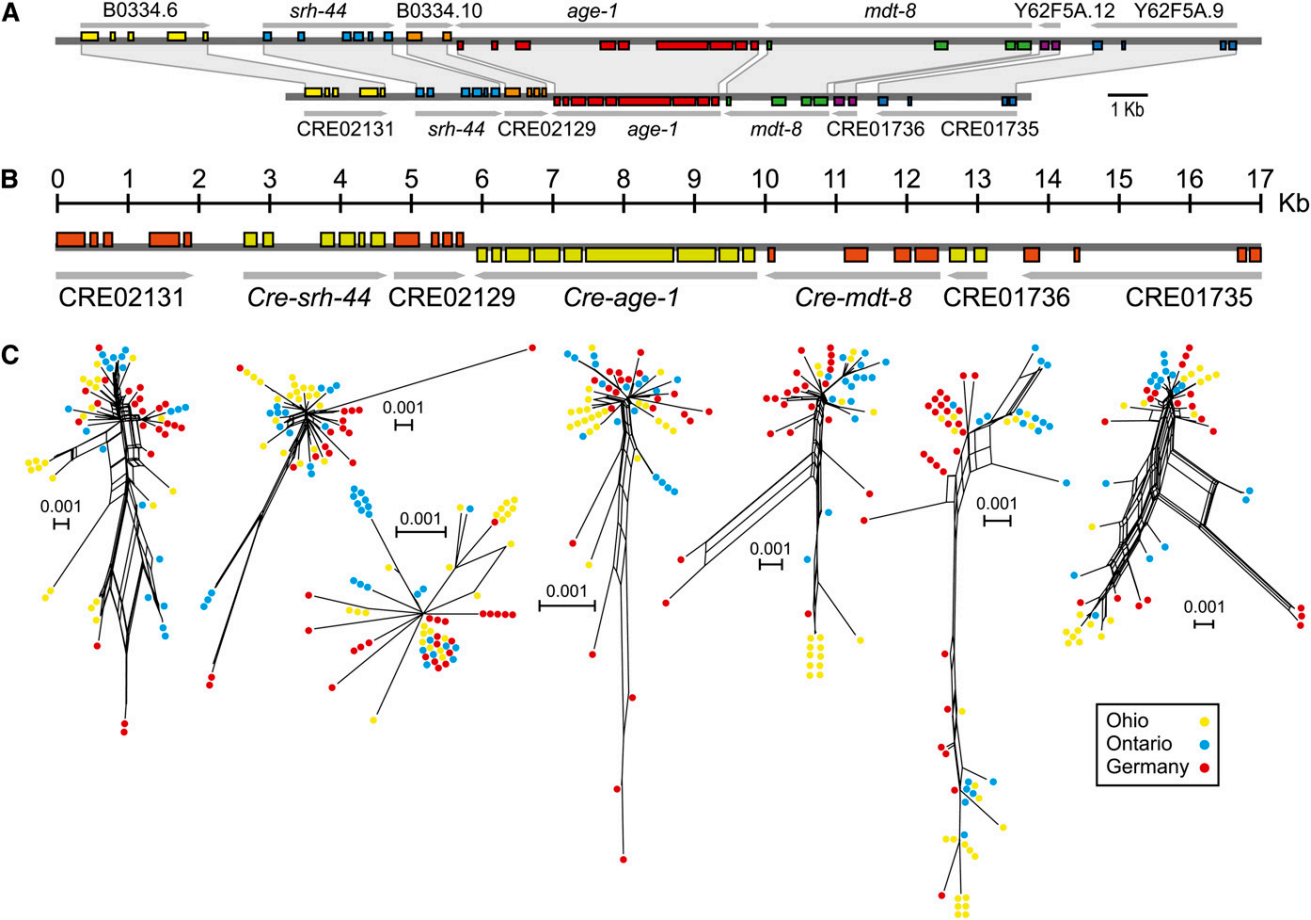
(A) Conserved synteny between *C. elegans* (top) and *C. remanei* (bottom) in the genomic region surrounding *age-1*. The structure is shown for each locus with boxes representing exons and is color-coded to show orthologous relationships. Arrows indicate orientation. (B) Genomic organization of *age-1* and its immediate neighbors. Each gene was resequenced in three populations of *C. remanei* to investigate patterns of diversity and selection in this region. (C) Neighbor-joining networks showing the relationships among *C. remanei strains*, color-coded according to their population of origin. A small number of strains with short internal branches are omitted for easier representation. Reticulation indicates potential recombination among strains.

*C. remanei* orthologs of the *C. elegans* insulin-signaling genes (highlighted in Figure 1) were identified from the current *C. remanei* genome assembly (version 15.0.1; Genome Sequencing Center, Washington University, St Louis, unpublished data) using the TBLASTN program (Altschul *et al.* 1990). Intron/exon boundaries were predicted with respect to the *C. elegans* protein sequence. No ortholog of *akt-2* could be identified, as it appears to be a gene duplication within the *C. elegans* lineage (Jovelin and Phillips 2011). Although some conserved exons could be identified, no clear ortholog of *daf*-18 could be found, presumably because of extensive divergence at this locus (see also Alvarez-Ponce *et al.* 2009). This procedure also was applied to the identification of the orthologs of genes immediately flanking *age-1*, which show conserved synteny between *C. elegans* and *C. remanei* (Figure 2A). Orthologs of *age-1* and its immediate neighbors were identified in *Caenorhabditis* sp. 23 through direct sequencing using *C. remanei*-specific primers. We obtained the full sequence for the *C*. sp. 23 orthologs of *age-1*, *srh-44*, *mdt-8*, CRE01736, CRE02129, and CRE02131 and partial sequence for the *C*. sp. 23 ortholog of CRE01735 (27%).

### Strains, amplification, and sequencing

The *C. remanei* strains used in this study are isofemale lines derived from individuals collected from isopods or decaying vegetal matter and sampled from three different populations in Dayton, Ohio; Kiel, Germany; and King City, Ontario, Canada (Cutter 2008; Jovelin *et al.* 2009; Dey *et al.* 2012). We also used a strain of the closely related species *Caenorhabditis* sp. 23, isolated from Wuhan City, China, as an outgroup (Dey *et al.* 2012). All strains were maintained on agar plates seeded with *Escherichia coli* OP50 following standard protocols (Brenner 1974).

For the *C. remanei* strains from Ohio, total RNA was extracted from plates containing individuals at all stages of development using the TRI Reagent protocol (Molecular Research Center) and subsequently used to synthesize double-stranded complementary DNA with the Retroscript kit (Ambion). Primers designed from the *C. remanei* genomic sequence were then used to amplify and sequence the coding region of the insulin signaling genes. DNA also was amplified from a single individual using the manufacturer’s protocol of the Repli-G kit (QIAGEN) for each strain of *C. remanei* from Ohio, *C. remanei* from Germany, and *C*. sp. 23. Genomic DNA isolated from a single individual was diluted 20 times before undergoing polymerase chain reaction. For each strain of *C. remanei* from Ontario, DNA was isolated from large populations of worms using the DNeasy Blood and Tissue kit (QIAGEN). Genomic DNA was then used as a template to amplify and sequence the coding and intronic regions of *age-1* and its three immediate neighbors in the 5′ and 3′ flanking regions (Figure 2). Amplifications were processed in 50-μL reaction volumes with 2.5 μL of dimethyl sulfoxide, 5 μL olf 10X Buffer (Fermentas), 4 μL of MgCl_2_, 0.6 μL of each primer (50 μM), 0.3 μL of TrueStart Taq polymerase (Fermentas), and 1 μL of template complementary DNA or 2 μL of genomic DNA. Cycling conditions were: 95° for 4 min followed by 35 cycles of 95° for 1 min, 55° or 58° for 1 min, and 72° for 3 min. Amplifications were sequenced using automated sequencers at the University of Oregon and University of Arizona sequencing facilities. All sequence changes were rechecked visually against sequencing chromatograms. Heterozygote sites were coded according to the International Union of Pure and Applied Chemistry nomenclature. Haplotypes were resolved using the program PHASE 2.1 (Stephens *et al.* 2001), implemented in DnaSP 5.10 (Librado and Rozas 2009). Both haplotypes were used for each strain in subsequent analyses.

### Relationships among strains and sampling scheme

We examined the relationships among strains by using neighbor networks generated with a Jukes-Cantor distance in the program SplitsTree 4.10 (Huson and Bryant 2006). We performed all population genetic analyses by using several sampling schemes: first, considering each population separately and second, grouping all strains together (Cutter *et al.* 2012).

### Nucleotide diversity and tests of neutrality

Insertions and deletions were excluded in all analyses. Estimates of nucleotide diversity (π; Nei 1987) were computed for different categories of sites with DnaSp 5.10 (Librado and Rozas 2009). The sliding window analysis of nucleotide diversity across the 17-Kb genomic region was performed using 673 windows, each 150 bp-long with a 25-bp step size.

We tested deviation from neutrality by using Tajima’s *D* (Tajima 1989) computed either using synonymous or silent (synonymous + intronic) sites. The significance of Tajima’s *D* was determined by coalescent simulations using DnaSP 5.10 with 50,000 replicates, making the conservative assumption of no intragenic recombination (Tajima 1989; Wall 1999) and conditioning on the number of segregating silent sites *S*. We combined our data with published data on polymorphism in the coding sequence of 87 genes with various function, sampled in the Ohio population, to plot the empirical distribution of Tajima’s *D* (Jovelin *et al.* 2003; Cutter *et al.* 2006; Cutter 2008; Jovelin 2009; Jovelin *et al.* 2009). We used *C*. sp. 23 as an outgroup to determine ancestral and derived alleles within our *C. remanei* samples (Dey *et al.* 2012). We then further tested deviations from neutral expectations by using the normalized Fay and Wu’s *H* statistics (Fay and Wu 2000; Zeng *et al.* 2006), and assessed significance by coalescent simulations with 10,000 replicates using the program DH (Zeng *et al.* 2006). Because the *H* test is sensitive to misidentification of ancestral and derived states, we estimated the probability of misorientation following the method developed by (Baudry and Depaulis 2003).

We used pairwise Hudson-Kreitman-Aguadé (HKA) tests (Hudson *et al.* 1987) and coalescent simulations with 10,000 replicates using the program HKA (J. Hey, unpublished data) to examine the significance of silent site nucleotide differences between *age-1* and its neighbors (Obbard *et al.* 2011). We also used maximum likelihood HKA tests (Wright and Charlesworth 2004) to further investigate patterns of selection at *age-1* and test the significance of the observed low level of neutral site nucleotide diversity. For this analysis, we combined our data with published polymorphisms at synonymous sites for 20 loci sampled in the same populations and for which the *C*. sp. 23 ortholog is available (Dey *et al.* 2012). Maximum likelihood estimates of *θ* and *k*, the selection parameter, were generated using 200,000 chains and with starting values of the parameters *T* and *θ* obtained by analyzing the data with the program HKA as described previously. We repeated this procedure three times to ensure that parameter estimates were similar. We performed a likelihood ratio test between the null hypothesis of neutral evolution and the alternative hypothesis of selection at *age-1*, and obtained significance of the likelihood ratio statistics 2ΔL by comparison with the χ^2^ distribution with 1 degree of freedom (Wright and Charlesworth 2004).

### Scans of selective sweep

We used the program SweepFinder (Nielsen *et al.* 2005) to test for a selective sweep in the vicinity of *age-1*. This method computes a likelihood ratio test between a model of a selective sweep to a null model obtained from the background frequency spectrum in the data. The grid size parameter was set to 125. We used the unfolded site frequency spectrum (SFS) with derived alleles determined by comparison with *C*. sp. 23 and used the folded SFS at sites where data are missing in *C*. sp. 23 or when the *C*. sp. 23 allele was distinct from the *C. remanei* alleles. To evaluate how missing data in intergenic regions between the genes of interest might affect our results, we resequenced the entire 17-Kb region, including intergenic sequence, in 15 individual worms from the population in Ohio. For this analysis, we performed the selective scan with SweepFinder using the folded SFS. In addition, we performed another sweep scan using patterns of LD with this dataset. This method identifies selected regions that are flanked by high LD but with low LD across the region (Kim and Nielsen 2004). We used the program OmegaPlus (Alachiotis *et al.* 2012) to compute the *ω* statistics describing this LD pattern under a selective sweep. The grid size parameter was set to 125, and the minwin and maxwin parameters were set, respectively, to 1000 bp and 2000 bp. For each analysis, the 1% cutoff value of the composite likelihood ratio (CLR) test and the *ω* statistics was obtained by coalescent simulations under the standard neutral equilibrium model with 10,000 replicates using the program ms (Hudson 2002). The standard neutral model provides a conservative test (Nielsen *et al.* 2005) and the pattern of polymorphism in *C. remanei* suggests demographic equilibrium, in particular in the populations from Ohio and Ontario (Cutter *et al.* 2006; Dey *et al.* 2012).

### Protein sequence divergence

The protein sequences of *C. remanei* and *C*. sp. 23 orthologs of each gene within the *age-1* genomic region were aligned by eye using BioEdit (Hall 1999) and subsequently used to generate codon-based DNA sequence alignments. Maximum likelihood estimates of the rates of nonsynonymous (*dN*) and synonymous (*dS*) substitutions were then computed between *C. remanei* and *C*. sp. 23 with the CODEML program in PAML 3.14 (Yang 1997). We examined adaptive evolution in the protein sequences of *age-1* and its neighbors by contrasting polymorphism and divergence in their coding sequence using the McDonald-Kreitman test (McDonald and Kreitman 1991).

## Results

### Patterns of variation across the IS pathway

We quantified nucleotide variation in the coding sequence of the IS genes in a population of *C. remanei* from Ohio to investigate the microevolution of insulin-signaling (Table 1). Overall levels of nucleotide variability are similar to previous reports in this species (Graustein *et al.* 2002; Jovelin *et al.* 2003; Haag and Ackerman 2005; Cutter *et al.* 2006; Cutter 2008; Jovelin 2009; Jovelin *et al.* 2009; Dey *et al.* 2012) with the key exception of the pattern of polymorphism at the *age-1* locus. There is no evidence that expression level (Spearman’s *ρ* = −0.071, *P* = 0.879) or pathway position (Spearman’s *ρ* = -0.132, *P* = 0.754) affect synonymous site diversity across the pathway as a whole (see also Jovelin and Phillips 2011). Nucleotide diversity at *age-1* is 20-fold lower than nucleotide diversity for the most polymorphic IS gene, *aap-1*, such that *age-1* has only 34 polymorphisms in 3564 bp of coding sequence (Table 1). More intriguing is the unusually low variation at *age-1* synonymous sites (π_s_ = 0.257%) relative to the other 7 IS genes (average π_s_ = 3.93%) and to other loci sampled in the same population (*n* = 91, average π_s_ = 3.75%). This low nucleotide diversity could result from a selective sweep linked to *age-1* or from strong purifying selection at synonymous sites.

**Table 1:** Pattern of nucleotide polymorphism in the coding sequence of the insulin-signaling genes in the Ohio population of *C. remanei*

Locus	Chr	N	n	% Seq	NS	P	A	S	π	π_a_	π_s_
*daf-2*	III	11	22	35	1941	60	9	52	10.32	1.87	38.82
*ist-1*	X	9	18	92	2749	40	13	27	4.25	1.62	13.37
*aap-1*	I	15	30	84	1306	96	12	83	18.41	2.23	72.49
*age-1*	II	24	48	100	3564	34	9	25	0.92	0.44	2.57
*pdk-1*	X	11	22	95	1791	76	6	70	14.81	1.76	59.34
*akt-1*	V	14	28	79	1456	42	2	40	11.08	0.88	46.93
*sgk-1*	X	12	24	88	1220	18	1	17	5.49	0.09	25.18
*daf-16*	II	13	26	93	1469	20	2	18	4.94	0.57	18.96
*sir-2.1*	IV	14	14	94	1715	78	21	60	14.03	3.73	49.81

π values are ×10^3^. Chr, chromosome in *C. elegans*; N, number of strains, n, number of chromosomes, twice the number of the strains when heterozygote sites were present; % Seq, percent of the *C. remanei* coding sequence analyzed; NS, number of sites analyzed (excluding gap positions); P, number of polymorphic sites; A, number of amino acid replacement changes; S, number of synonymous changes; π, total nucleotide diversity; π_a_, nucleotide diversity at nonsynonymous sites; π_s_, nucleotide diversity at synonymous sites.

### A recent selective sweep at the *age-1* locus

**Figure 3:**
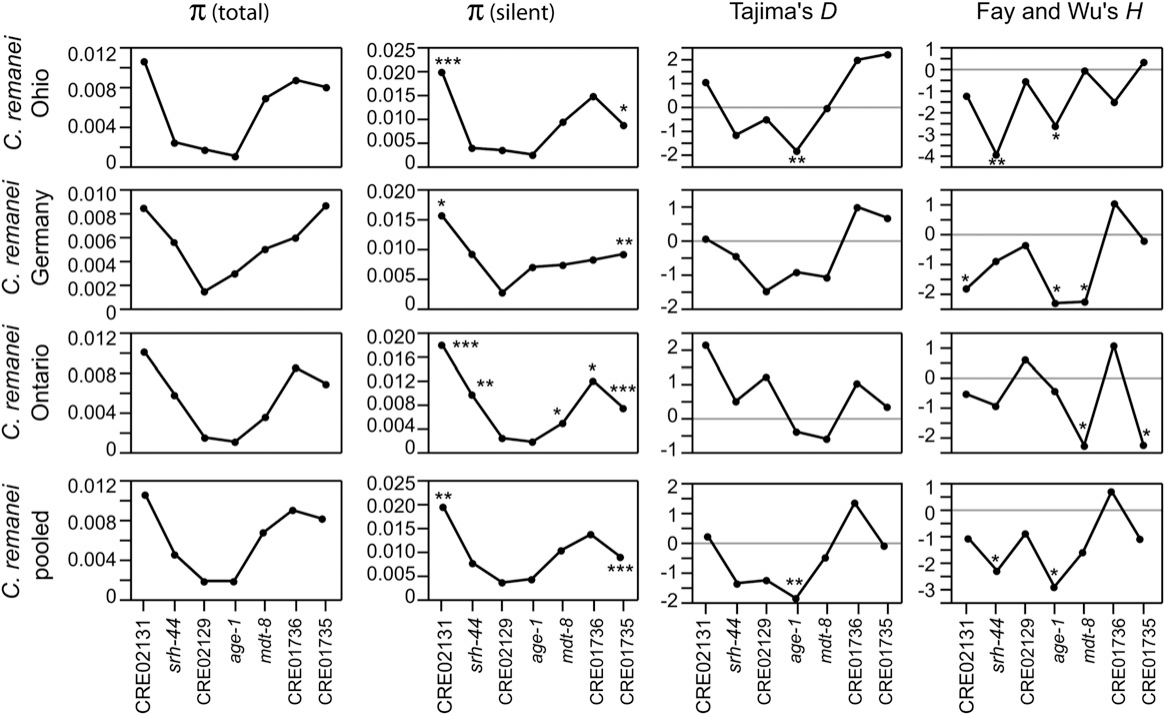
The nucleotide diversity around *age-1* is reduced in three populations of *C. remanei* and at a global spatial scale. The site frequency spectrum shows an excess of rare alleles (Tajima’s *D*) and an excess of derived high-frequency variants (Fay and Wu’s *H*) localizing directly at *age-1* and/or on its close neighbors. Significance of the difference in silent site nucleotide diversity between *age-1* and each of its neighbors was assessed using pairwise HKA tests. Significance of the Tajima’s *D* and Fay and Wu’s *H* statistics were determined by coalescent simulations. *P < 0.05, **P < 0.01, ***P < 0.001.

Natural selection can be uncovered because of the signatures it leaves in the genomic sequence around the sites under selection. A selective sweep results in a reduction of nucleotide diversity because linked neutral variants hitchhike with the selected allele (Maynard Smith and Haigh 1974). To test for such an effect on *age-1*, we collected polymorphism data in the coding and intronic regions of *age-1* and its three upstream and downstream immediate neighbors, located within a 17-kb region, from three populations of *C. remanei* (Ohio, Ontario, and Germany, Table 2 and Figure 2). In addition, we sequenced the orthologs of these seven genes in the closely related species *Caenorhabditis* sp. 23 (Dey *et al.* 2012) to measure interspecific sequence divergence and to polarize the ancestry of polymorphisms within *C. remanei*. There is a clear reduction of nucleotide diversity centered directly on *age-1* in the populations from Ohio and Ontario and centered on CRE02129 in the population from Germany (Figure 3). We combined data from the three populations to examine global patterns of nucleotide variation within *C. remanei*. Similarly, nucleotide polymorphism is lowest for *age-1* and CRE02129 in the pooled sample and increases as a function of the distance from these two genes (Figure 3).

**Table 2:** Pattern of nucleotide variation at *age-1* and at its immediate upstream and downstream neighbors in three populations of *C. remanei*

Sample	Locus	N	n	% Seq	NS	P	A	S	π	π_a_	π_s_	π_si_	*D_Taj_*	*H_FW_*
Ohio	CRE02131	22	44	100	1860	66	3	23	10.66	0.76	29.24	19.97	1.069	−1.208
	*srh-44*	25	50	100	1905	33	2	8	2.46	0.20	5.83	4.03	−1.150	−3.924**
	CRE02129	24	24	100	961	8	1	1	1.73	0.16	1.40	3.58	−0.498	−0.536
	*age-1*	24	48	100	3961	39	9	25	1.09	0.44	2.57	2.56	−1.821*	−2.604*
	*mdt-8*	17	17	100	2448	57	3	7	6.94	1.44	8.52	9.51	−0.024	−0.041
	CRE01736	22	44	100	509	11	4	3	8.77	5.19	15.23	14.89	2.004	−1.492
	CRE01735	19	38	98	3371	70	2	3	8.05	2.50	13.80	8.82	2.236	0.354
Germany	CRE02131	22	44	100	1869	68	3	20	8.51	0.74	17.90	15.76	0.073	−1.804*
	*srh-44*	23	46	100	1916	54	1	13	5.62	0.30	9.95	9.28	−0.440	−0.882
	CRE02129	24	48	100	960	14	3	3	1.50	0.39	1.74	2.82	−1.470	−0.347
	*age-1*	24	48	100	3959	71	18	38	3.00	1.23	8.24	7.08	−0.910	−2.293*
	*mdt-8*	23	46	68	1682	55	9	12	5.15	1.77	9.15	7.43	−1.067	−2.244*
	CRE01736	25	50	100	510	13	8	11	6.01	4.63	9.67	8.34	0.999	1.052
	CRE01735	19	38	99	3393	108	11	11	8.72	5.14	27.29	9.25	0.681	−0.201
Ontario	CRE02131	19	38	100	1869	51	5	17	10.16	1.67	23.71	18.08	2.167	−0.515
	*srh-44*	17	34	100	1922	40	0	8	5.82	0	10.32	9.79	0.516	−0.926
	CRE02129	21	42	100	960	5	2	1	1.54	0.72	3.08	2.50	1.233	0.625
	*age-1*	20	40	100	3961	19	8	9	1.12	0.79	2.21	1.88	−0.376	−0.423
	*mdt-8*	17	34	73	1802	33	6	5	3.62	1.46	4.59	5.02	−0.586	−2.262*
	CRE01736	19	38	100	510	13	4	5	8.57	6.50	14.66	12.09	1.039	1.092
	CRE01735	17	34	98	3374	87	4	5	6.92	2.73	14.79	7.52	0.355	−2.233*
Pooled	CRE02131	63	126	100	1855	101	8	30	10.62	1.20	25.98	19.54	0.237	−1.062
	*srh-44*	65	130	100	1901	83	2	17	4.59	0.19	9.11	7.65	−1.350	−2.287*
	CRE02129	69	138	100	960	22	6	4	1.88	0.43	2.52	3.59	−1.233	−0.873
	*age-1*	68	136	100	3959	94	23	53	1.89	0.87	4.73	4.24	−1.839**	−2.897*
	*mdt-8*	57	114	68	1682	76	13	15	6.82	1.95	9.51	10.35	−0.472	−1.582
	CRE01736	66	132	100	509	20	9	5	9.08	6.33	15.29	13.77	1.367	0.716
	CRE01735	55	110	97	3330	148	6	11	8.21	2.66	20.08	8.96	−0.076	−1.078

Tajima’s *D* was computed using silent sites diversity. π values are ×10^3^. N, number of strains; n, number of chromosomes, twice the number of the strains when heterozygote sites were present; % Seq, percent of the *C. remanei* gene sequenced; NS, number of sites analyzed (excluding gap positions); P, number of polymorphic sites; A, number of amino acid replacement changes; S, number of synonymous changes; π, total nucleotide diversity; π_a_, nucleotide diversity at nonsynonymous sites; π_s_, nucleotide diversity at synonymous sites; π_si_, nucleotide diversity at silent sites.

* *P* < 0.05.

** *P* < 0.01.

We then performed pairwise HKA tests between *age-1* and each of its neighbors to determine the significance of the reduction of nucleotide diversity at *age-1* (Hudson *et al.* 1987). In all population samples, the nucleotide diversity at silent sites is significantly reduced at *age-1* relative to its two most distant neighbors, and in the Ontario population all genes but CRE02129 have significantly higher silent site nucleotide variation than *age-1* (Figure 3). To further explore selection at *age-1*, we contrasted multilocus polymorphism and divergence by combining our data with a larger set of genes (Dey *et al.* 2012) and used the maximum likelihood HKA framework (Wright and Charlesworth 2004). Synonymous site variation in *age-1* is significantly reduced relative to the neutral model in all samples but the German population, consistent with the action of positive selection (Table 3).

**Table 3:** HKA likelihood ratio tests of selection at *age-1*

Sampling scheme	Hypothesis	*k* (*age-1*)	*L*	*2ΔL*	*P*
*C. remanei* - Ohio	No selection	1	−186.32		
	Selection	0.286	−182.27	8.10	0.0044
*C. remanei* – Germany	No selection	1	−161.11		
	Selection	0.448	−160.11	1.16	0.2815
*C. remanei* – Ontario	No selection	1	−186.4		
	Selection	0.096	−177.17	18.46	< 0.0001
*C. remanei* - pooled	No selection	1	−178.76		
	Selection	0.516	−175.98	5.56	0.0184

*k*, selection parameter, *k* < 1 indicates a reduction in diversity due to selection; *L*, log-likelihood of the hypothesis; *2ΔL*, likelihood ratio statistics.

**Figure 4:**
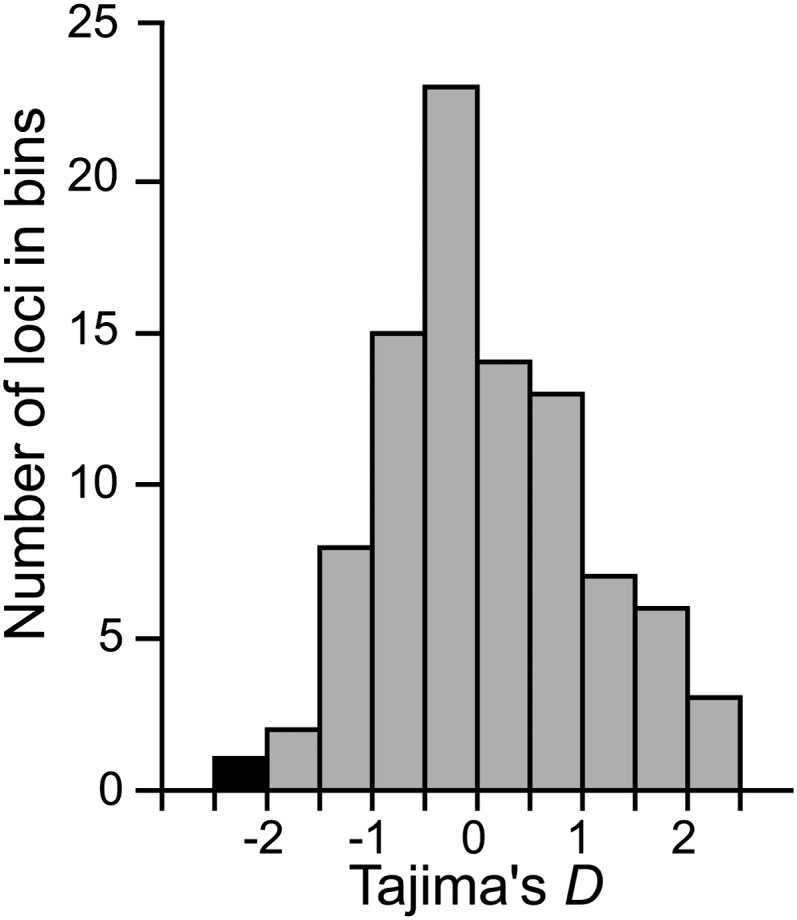
Empirical distribution of Tajima’s *D* from 92 protein coding genes sequenced from the same population in Ohio. *Cre-age-1* has the most negative Tajima’s *D* value (black bar), suggesting that the excess of rare variants at *Cre-age-1* is not the result of genome-wide demographic effects. Tajima’s *D* was computed using synonymous site diversity.

A selective sweep perturbs the SFS such that it results in an excess of low-frequency variants at linked sites (Tajima 1989). Thus, we first quantified the SFS by using Tajima’s *D* (*D_Taj_*) (Tajima 1989). In the Ohio population and in the pooled sample, *D_Taj_* is significantly negative for *age-1* but not for its neighbors (Figure 3). Moreover, the number of rare alleles decreases a function of the distance from *age-1*, suggesting that *age-1* is the focal point of a selective sweep (Figure 3). *D_Taj_* values also form a valley in the populations from Germany and Ontario, with negative values for *age-1* and its closest neighbors, although genes with the most negative values are the immediate neighbors CRE02129 in the German population and *mdt-8* in the Ontario population (Figure 3). Demographic factors, such as population growth, also can result in an excess of low-frequency alleles and significant *D_Taj_* values across the entire genome. However, we found that the value of *D_Taj_* for *age-1* is the most negative among 92 protein-coding genes, indicating that demographic history is insufficient to explain the strong skew in SFS for *age-1* (synonymous sites, *D_Taj_* = -2.08, *P* < 0.01, Figure 4).

Alternatively, the reduction of nucleotide diversity we observed around *age-1* could be the result of background selection, the removal of neutral variants linked to deleterious mutations (Charlesworth *et al.* 1993), and so negative *D_Taj_* values may reveal purifying selection (Tajima 1989). However, another signature of a selective sweep is an excess of derived high-frequency variants (Fay and Wu 2000). Fay and Wu’s *H* (*H_FW_*) is significantly negative for *age-1* in the Ohio, German and pooled samples, indicating that *age-1* has an excess of derived high-frequency single-nucleotide polymorphisms relative to neutral expectations (Figure 3). However, other genes also have significant negative values of *H_FW_*, depending on the sampling scheme, suggesting that the SFS at these genes is somewhat perturbed by the sweep (Figure 3). A potential issue associated with the *H* test is the misidentification of ancestral and derived states as the *H* test is very sensitive to homoplasy (Baudry and Depaulis 2003). Nevertheless, our results are unlikely to be an artifact of misorientation because the inferred probability of misorientation in our data are 0.078% (0.062% for *age-1*) (Baudry and Depaulis 2003). Altogether, the patterns of polymorphism and SFS suggest that *age-1* is the direct target or is tightly linked to a target of a selective sweep that affects *C. remanei* on a global spatial scale.

### Selective sweep scans

**Figure 5:**
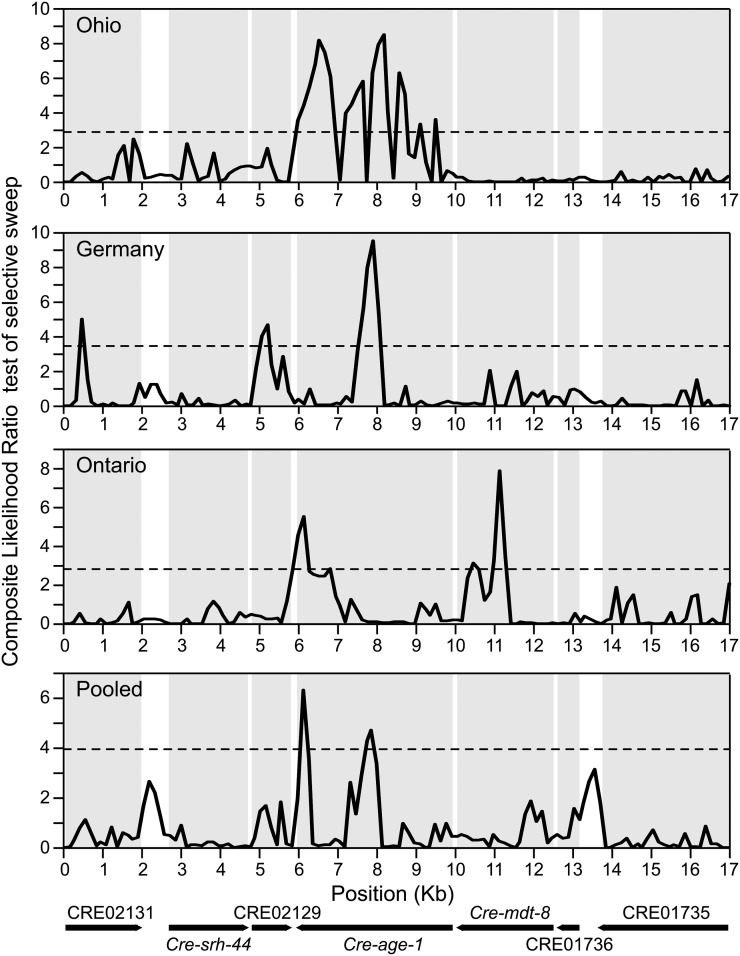
Composite likelihood ratio (CLR) test of selective sweep for genes in the genomic vicinity of the *age-1* locus. The CLR suggests that *age-1* is the direct target of a selective sweep in all three populations of *C. remanei*. The horizontal dashed lines indicate the 1% cut-off value as determined by simulations under the standard neutral model. The position of each gene within the 17-kb region is shown at the bottom and with gray boxes in each panel.

We used the method of (Nielsen *et al.* 2005) to scan for a selective sweep within the *age-1* genomic region. This method performs a likelihood ratio test between a model of selective sweep and a null model derived directly from the observed SFS in the data. The CLR is maximized and is significant (*P* < 0.01) at *age-1* in all three populations and in the pooled sample, although the exact position of CLR_max_ and the shape of the likelihood ratio surface vary between samples (Figure 5). For the Ontario population, the CLR also is significant for *mdt-8*, consistent with the analyses of the SFS based on Tajima’s *D* and Fay and Wu’s *H* (Figure 3). Thus, these analyses further implicate *age-1* as the target of a global selective sweep.

**Figure 6:**
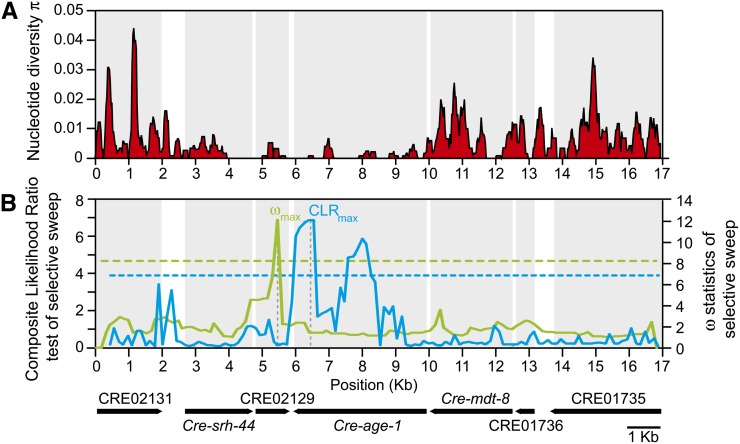
(A) A sliding window of nucleotide diversity of genic and intergenic regions identifies a ~6-kb genomic region, including CRE2129 and *Cre-age-1*, with low polymorphism. (B) The composite likelihood ratio (CLR) test of selective sweep based on the SFS is shown in blue. The CLR is maximized within *Cre-age-1*. The distribution of the *ω* statistics detecting a selective sweep based on the pattern of linkage disequilibrium is shown in green. The *ω* is maximized within CRE02129. Both tests identify a narrow region as the target of a selective sweep. The horizontal dashed lines indicate the 1% cut-off value as determined by simulations under the standard neutral model. The position of each gene within the 17-kb region and scaled with the *x* axes of (A) and (B) is shown at the bottom and with gray boxes in each panel.

All the aforementioned results for the 17-kb region encompassing *age-1* its neighbors are based on polymorphisms collected in the coding and intronic sequences of these genes. Although intergenic sequence comprises only ~11% of this genomic region and is thus unlikely to affect our results, we nevertheless sequenced the entire 17-kb region, including intergenic sequence, in 15 individuals from the Ohio population and re-examined signatures of selective sweep with this data. First, consistent with the pattern of diversity at individual loci, a sliding window analysis shows a clear reduction in nucleotide diversity within a ~6-kb region spanning from the end of *shr-44* through CRE02129 to *age-1* (Figure 6A). Second, the CLR along the genomic region is maximized within *age-1* at position +3391 (relative to the start codon) in exon 7 (Figure 6B, CLR_max_ = 6.84, *P* < 0.01). Third, we further examined the occurrence of a selective sweep in the *age-1* genomic region using patterns of LD. Another signature of a selective sweep is increased LD on each side but low LD across the selected region (Kim and Nielsen 2004; Pavlidis *et al.* 2010; Alachiotis *et al.* 2012). The *ω* statistics, measuring the LD pattern under a sweep, is maximized within CRE02129 at position +913 (relative to start codon) in the last exon (Figure 6B, *ω*_max_ = 12.19, *P* < 0.01). Both methods of selective sweep detection based on the SFS and LD identify a narrow selected region as the CLR_max_ and *ω*_max_ are distant from each other by only ~1 kb (Figure 6B). Because we targeted the *age-1* region for further analysis based on our findings for the different components of the IS pathway, there is the possible concern of statistical ascertainment bias (Thornton and Jensen 2007). However, such an issue should be less pronounced for our *a priori* selected pathway scan than for a full genome scan (which leads to numerous posthoc tests), and the *P*-values associated with our analysis of the *age-1* region suggest that statistical significance of our findings will be robust to moderate adjustment of the significance threshold (Supporting Information, Table S1).

### Protein sequence divergence of *age-1* and its neighbors

The models of selective sweep based on LD and the SFS are most powerful in detecting recent hitchhiking events (Nielsen *et al.* 2005; Pavlidis *et al.* 2010). To investigate selection over longer evolutionary time scales in the coding sequence of *age-1* and its neighbors, we contrasted patterns of polymorphism within species with sequence divergence between species using the McDonald-Kreitman test (McDonald and Kreitman 1991). First, we note that CRE02136 and *age-1* have the highest *dN/dS* values among the 7 genes tested, indicating relatively rapid protein sequence divergence (Table 4). Second, we found that the ratios of non-synonymous to synonymous polymorphisms and substitutions are not equal for CRE02129 and *age-1*, as would be expected by the Neutral Theory (Table 4). CRE02129 exhibits long term purifying selection with a significant deficit of sequence divergence (*P* = 0.001). However, *age-1* shows a significant excess of sequence divergence relative to polymorphism (*P* = 0.037), implicating repeated fixation of adaptive mutations by positive selection in its coding sequence. Altogether, our results strongly support *age-1* as the focal point of positive directional selection and a global selective sweep.

**Table 4:** Sequence evolution and MK tests of adaptive divergence

Locus	*dN*	*dS*	*dN*/*dS*	Da	Ds	Da/Ds	Pa	Ps	Pa/Ps	*P*
CRE02131	0.0012	0.0829	0.0145	1	16	0.0625	8	31	0.2581	0.2502
*srh-44*	0.0027	0.0887	0.0304	2	17	0.1176	2	17	0.1176	1
CRE02129	0.0019	0.1028	0.0185	0	15	0	6	4	1.5	0.0012
*age-1*	0.0318	0.1404	0.2265	79	94	0.8404	24	53	0.4528	0.0369
*mdt-8*	0.0118	0.1098	0.1075	5	18	0.2778	13	16	0.8125	0.1415
CRE01736	0.0514	0.1223	0.4203	11	10	1.1	9	5	1.8	0.7282
CRE01735	0	0.0089	0	0	1	0	4	3	1.3333	1

MK, McDonald-Kreitman; Da, fixed amino acid replacements; Ds, fixed synonymous changes; Pa, nonsynonymous polymorphisms; Ps, synonymous polymorphisms.

## Discussion

Evolutionary theories of aging predict that senescence evolves as a result of a trade-off between maintenance and repair of the soma and investment in reproduction (Kirkwood 2002). In most circumstances, reproduction that occurs earlier in life will have a larger effect on fitness and on the rate of population growth than reproduction that occurs later in life (Rose *et al.* 2008). Thus, under the antagonistic pleiotropy theory of aging, beneficial mutations early in life will be favored even if they cause deleterious effects late in life (Willams 1957). If existing genetic systems have evolved under these conditions, then we would expect mutations that increase lifespan to have negative effects on reproduction (and vice versa). Both the insulin-like receptor *daf-2* and the phosphatidylinositol 3-OH kinase (PI3K) catalytic subunit *age-1*, which are known to increase lifespan when mutated (Kimura *et al.* 1997; Ayyadevara *et al.* 2008), exhibit a fitness cost under nutrient stress, as predicted by the antagonistic pleiotropy model (Walker *et al.* 2000; Jenkins *et al.* 2004). However, all of these studies have been conducted with the use of induced mutations whose effects have been examined under laboratory conditions (although see Van Voorhies *et al.* 2005). In nature we might expect the optimal balance between reproduction and somatic maintenance to shift depending on environmental conditions and local demography. Further, natural allelic variation may or may not well represent the severe effects displayed by mutations isolated and studied in the laboratory (Anderson *et al.* 2011). How then, does natural selection shape variation in these genetic pathways in nature?

### The PI3K catalytic subunit *age-1* is the target of a recent selective sweep in *C. remanei*

Our analysis of DNA sequence variation in the IS pathway shows that polymorphism at most loci is high and very similar to that observed in other genes with a wide range of biological functions (Jovelin *et al.* 2003; Cutter *et al.* 2006; Cutter 2008; Jovelin 2009; Jovelin *et al.* 2009). However, variation in one gene, the *age-1* PI3K, is much lower than any other gene in the pathway and, indeed, is lower than any other previously examined locus within this species. Analysis of a broader distribution of polymorphism in multiple populations clearly demonstrates that this region of the genome has recently undergone a global selective sweep that appears to be centered directly at the *age-1* locus.

Although a comparative analysis among species within the *Caenorhabditis* genus has shown that divergence among IS pathway components appears to be largely driven by differences in gene expression (Jovelin and Phillips 2011), we do not see this pattern reflected in within-population variation. In *C. elegans*, *age-1* is part of an operon that includes genes *mdt-8* and Y62F5A.12. More generally, *age-1* is located in a highly compact genomic region in which the distance between two gene neighbors is only a few hundred base-pairs long (Figure 2). We detected strong purifying selection on CRE02129, the closest downstream neighbor of *age-1*. However, the pattern of diversity at *age-1* does not result from linked negative selection at CRE02129. Explicit models consistently localize *age-1* as a target of a selective sweep. Moreover, the abundance of high-frequency derived single-nucleotide polymorphisms and the rapid protein sequence divergence in *age-1* are not compatible with background selection shaping diversity within this gene. Nevertheless, the short distance between *age-1* and CRE02129 invites the question of how positive and negative selection interfere in this genomic region (Hill and Robertson 1966).

The function of AGE-1 is to convert phosphatidylinositol(4,5)P_2_ into phosphatidylinositol(3,4,5)P_3_. Membrane-bound phosphatidylinositol(3,4,5)P3 then recruits the IS kinases PDK-1, AKT-1, and SGK-1, as well as presumably many other signal-transduction proteins that possess a pleckstrin-homology domain (Shmookler Reis *et al.* 2009). Loss of function mutations in *age-1* not only affect overall kinase activity but also down-regulate the transcription of several genes in the IS pathway as well as in other signaling-pathways (Tazearslan *et al.* 2009). Mutations in *age-1* have high potential to induce broad regulatory effects that affect fitness even beyond its well-studied role in stress response and aging. Thus, although *age-1* appears to be an ideal example of a gene in which a direct connection can be made between the mode of selection in natural populations and trade-off between increased fitness and senescence as predicted by the antagonistic pleiotropy model, tests of specific allelic function are needed to establish whether or not the pattern of selection detected here can be directly attributed to a trade-off between lifespan and reproduction. Interestingly, in a comprehensive comparative analysis of differences in gene expression over development between *C. elegans* and *C. briggsae*, Grün *et al.* (2014) found that expression for genes involved in the insulin-signaling pathway displayed the strongest signal of divergence across the entire genome, potentially indicating adaptive divergence within these species as well.

We did not detect unusual nucleotide diversity for *daf-2* (Table 1), although we surveyed only 35% of the *daf-2* coding sequence. However, it is noteworthy that positive selection has been detected in the *daf-2* ortholog in *Drosophila InR* (Guirao-Rico and Aguade 2009). Thus, *daf-2*/*InR* might provide another example of a gene with antagonistic pleiotropic effects on aging and reproduction that evolves by positive selection.

### *Caenorhabditis* as a model system for population genomics

This is the first report of a recent selective sweep localized to a targeted gene in *Caenorhabditis*. Low nucleotide variation and extensive LD make investigation of selected targets difficult in *C. elegans* (Cutter 2006; Jovelin *et al.* 2009; Rockman and Kruglyak 2009; Rockman *et al.* 2010; Andersen *et al.* 2012), particularly if the aim is to tie molecular variation to a specific evolutionary context. High nucleotide diversity in *C. remanei* (Cutter *et al.* 2013), coupled with the rapid decay of LD (Cutter *et al.* 2006), suggest that genome-wide scans will be successful in localizing targets of adaptive evolution in this species. Furthermore, *C*. *remanei* displays a great deal of genetic variation for a variety of phenotypes, including those associated with stress resistance and longevity (Reynolds and Phillips 2013). With the wealth of information on genetics, development and cell biology obtained from decades of research in *C. elegans* and the increasing availability of genomic resources from a number of different species, *Caenorhabditis* is rapidly joining *Drosophila* as an excellent model clade for evolutionary genetic analyses. Overall, then, we are now at a stage in which general theories regarding the evolution of biological systems as seemingly complex as aging can be directly tested by combining our rapidly expanding knowledge of the molecular function of critical pathways with comprehensive population genetic analyses of pathway components.

## Supplementary Material

Supporting Information
