# Supplementary material for: A Recent Global Selective Sweep on the age-1 Phosphatidylinositol 3-OH Kinase Regulator of the Insulin-Like Signaling Pathway Within Caenorhabditis remanei
Source: G3 (Bethesda). 2014 Apr 11;4(6):1123–33. doi: 10.1534/g3.114.010629 (PMC4065255; doi:10.1534/g3.114.010629)
Supplement: Supporting Information [file supp_4_6_1123__index.html]

A Recent Global Selective Sweep on the age-1 Phosphatidylinositol 3-OH Kinase Regulator of the Insulin-Like Signaling Pathway Within Caenorhabditis remanei — Supporting Information 

# A Recent Global Selective Sweep on the *age-1* Phosphatidylinositol 3-OH Kinase Regulator of the Insulin-Like Signaling Pathway Within *Caenorhabditis remanei*

## Supporting Information for Jovelin *et al.*, 2014

**Files in this Data Supplement:**

- Table S1 - *P*-values for neutrality tests for age-1 and its neighbors in different population samples. (PDF, 156 KB)
