# Supplementary material for: A Recent Global Selective Sweep on the age-1 Phosphatidylinositol 3-OH Kinase Regulator of the Insulin-Like Signaling Pathway Within Caenorhabditis remanei
Source: G3 (Bethesda). 2014 Apr 11;4(6):1123–33. doi: 10.1534/g3.114.010629 (PMC4065255; doi:10.1534/g3.114.010629)
Supplement: Supporting Information [file supp_g3.114.010629_010629SI.pdf]

G3: Genes | Genomes | Genetics

A recent global selective sweep on the *age-1* PI3 kinase regulator of the insulin-like signaling pathway within *Caenorhabditis remanei*

Richard Jovelin*[,†], Jennifer S. Comstock*, Asher D. Cutter[†], Patrick C. Phillips*[1]

*Institute of Ecology and Evolution, University of Oregon, Oregon 97403, USA
[†]Department of Ecology and Evolutionary Biology, University of Toronto, Ontario M5S 3B2

Sequences were deposited in GenBank under accession numbers JN251161-JN251322 and KF925534-KF925830

[1]Corresponding author: Patrick C. Phillips. Institute of Ecology and Evolution and Department of Biology, University of Oregon, Eugene, OR 97403-5289 USA; Email: pphil@uoregon.edu

DOI: 10.1534/g3.114.010629

Table S1 *P*-values for neutrality tests for age-1 and its neighbors in different population samples. D_{Taj}: Tajima's *D*; H_{FW}: Fay and Wu's *H*; HKA: pairwise Hudson-Aguade-Kreitman tests between *age-1* and each neighbor. NA: not applicable

	Ohio			Ontario			Germany			Pooled		
Locus	D_{Taj}	H_{FW}	HKA	D_{Taj}	H_{FW}	HKA	D_{Taj}	H_{FW}	HKA	D_{Taj}	H_{FW}	HKA
CRE02131	0.896	0.088	**0.001**	0.991	0.187	**0.000**	0.606	**0.044**	**0.038**	0.689	0.102	**0.013**
SRH-44	0.119	**0.003**	0.552	0.761	0.121	**0.008**	0.383	0.128	0.631	0.063	**0.027**	0.276
CRE02129	0.339	0.181	0.823	0.896	0.747	0.718	0.050	0.214	0.877	**0.094**	0.127	0.942
AGE-1	**0.013**	**0.019**	NA	0.414	0.202	NA	0.189	**0.024**	NA	**0.008**	0.012	NA
MDT-8	0.545	0.311	0.402	0.314	**0.028**	**0.027**	0.138	**0.027**	0.597	0.568	0.059	0.404
CRE01736	0.979	0.068	0.488	0.872	0.991	**0.015**	0.860	0.985	0.391	0.924	0.828	0.629
CRE01735	0.997	0.496	**0.018**	0.708	**0.029**	**0.000**	0.815	0.257	**0.003**	0.554	0.102	**0.001**
